# The value of plasma omega-3 polyunsaturated fatty acids in predicting the response and prognosis of cervical squamous cell carcinoma patients to concurrent chemoradiotherapy

**DOI:** 10.3389/fphar.2024.1379508

**Published:** 2024-05-27

**Authors:** Pengbin Ping, Juan Li, Xiaoying Xu

**Affiliations:** Department of Radiotherapy Oncology, The Second Affiliated Hospital of Dalian Medical University, Dalian, China

**Keywords:** cervical squamous cell carcinoma, concurrent chemoradiotherapy, omega-3 polyunsaturated fatty acids, eicosapentaenoic acid, response, prognosis

## Abstract

**Background:** In recent years, abnormalities in plasma omega-3 polyunsaturated fatty acids (omega-3 PUFAs) have been proven to be related to the risk of cancer, but their prognostic value for cancer is unclear. The purpose of this study was to retrospectively evaluate the response and prognostic significance of plasma omega-3 PUFAs in patients with cervical squamous cell carcinoma (CSCC) treated with concurrent chemoradiotherapy (CCRT). Spearman rank correlation analysis was used to analyze the correlation between omega-3 PUFAs and squamous cell carcinoma antigen (SCC-Ag) levels.

**Methods:** A total of 89 patients with CSCC who underwent CCRT were evaluated retrospectively. Binary logistic regression analysis was used to analyze the independent predictors related to complete response (CR) after CCRT. A Cox proportional hazard model and Kaplan-Meier analysis were utilized to perform survival analysis.

**Results:** According to multivariate logistic regression analyses, a high level of plasma EPA was independently correlated with an increased incidence of CR after CCRT (odds ratio (OR), 0.980; 95% confidence interval (CI), 0.962–0.999, *p* = 0.038). With a median follow-up of 41.3 months, the CSCC patients in the high EPA (≥46.0 nmol/mL) group exhibited longer OS and PFS. According to our multivariate analysis, pretreatment plasma EPA level was an independent prognostic factor for PFS in patients with CSCC who underwent CCRT (hazard ratio (HR), 0.263; 95% CI, 0.089–0.782, *p* = 0.016). However, it was not an independent prognostic factor of OS. Spearman rank correlation analysis revealed was a negative correlation between pretreatment SCC-Ag (pre SCC-Ag) levels and EPA levels (*r* = −0.305, *p* = 0.004), and a weak negative correlation between posttreatment SCC-Ag (post SCC-Ag) levels and EPA levels (*r* = −0.251, *p* = 0.018).

**Conclusion:** Plasma omega-3 PUFAs are related to the response and survival outcome of patients with CSCC who underwent CCRT. Pretreatment plasma EPA levels may be a promising biomarker for predicting the response and prognosis of patients with CSCC who undergo CCRT. In addition, the pretreatment plasma EPA levels presented a negative correlation with the SCC-Ag levels.

## 1 Introduction

Currently, According to GLOBOCAN2020 data, cervical cancer (CC) accounts for approximately 6.5% of all malignant tumors in women worldwide, making it the fourth most common cancer among women (Sung et al., 2021). Cervical squamous cell carcinoma (CSCC) and cervical adenocarcinoma are the most common subtypes of CC, accounting for 70% and 25% of all cases, respectively (Guo et al., 2018; Jenkins et al., 2020). It is undeniable that newer screening, awareness and technology have reduced the growth of CC in the past decade (Sung et al., 2021). Due to the low coverage of CC screening in middle-and low-income countries, a considerable number of patients are diagnosed with locally advanced cervical cancer (LACC) at the first diagnosis, including patients with stage IB3-IVA disease (Serkies and Jassem, 2018). Since 1999, cisplatin-based CCRT has become the standard treatment for patients with LACC, for which the total effective rate is more than 90% (Rose et al., 1999). However, approximately 30%–50% of patients with CC still experience local recurrence or distant metastasis after undergoing CCRT (Rose et al., 1999; Kumar and Gupta, 2016). Therefore, determining the prognostic factors of patients with LACC treated with CCRT is highly important.

Many studies have shown that PUFAs act vital roles in the occurrence of malignant tumors, including in the regulation of apoptosis (Haycock et al., 2023; Montecillo-Aguado et al., 2023). Omega-3 PUFAs are one of the polysaturated fatty acids, that are derived mainly from deep-sea fishes, such as sardines and herring, thus, they are also known as marine PUFAs (Montecillo-Aguado et al., 2023). It has been widely reported in epidemiological studies because of its remarkable anti-inflammatory effects, direct inhibition of cancer expansion and tumorigenesis (Vaughan et al., 2013; Wei et al., 2022). Omega-3 PUFAs mainly include eicosapentaenoic acid (C20:5, EPA), docosahexaenoic acid (C22:6, DHA) and docosapentaenoic acid (C22:5, DPA), among them, EPA and DHA have the highest biological activity. DPA are the intermediates of DHA and EPA and may also have potential anticancer properties (Vaughan et al., 2013).

Andrew et al. reported that EPA has significant anti-angiogenic effects on colorectal cancer, and that high levels of EPA could improve overall survival (OS) (Cockbain et al., 2014). A meta-analysis of 20 studies showed that omega-3 PUFAs significantly improved nutritional status and immune function in patients with colorectal cancer after radical resection after strict control of bias risk (Yue et al., 2022). Endogenous omega-3 PUFAs enrichment can reduce cisplatin-induced myelosuppression by regulating the NRF2-MDM2-p53 signaling pathway (Xu et al., 2023). Interestingly, the introduction of omega-3 PUFAs in esophageal, colorectal and breast cancer has been gradually proven to enhance the cytotoxicity of some chemotherapeutic drugs and enhance the sensitivity of tumors to ionizing radiation, but does not increase the toxicity to normal tissues (Cai et al., 2014; Shin et al., 2019; Yang et al., 2022). Kai and other researchers have showen that the unsaturation of exogenous fatty acids is positively correlated with ferroptosis sensitivity. DHA can effectively promote ferroptosis by mediating intracellular lipid peroxidation to kill CC HeLa cells (Shan et al., 2022). Supplementation with omega-3 PUFAs is effective at maintaining skeletal muscle mass, nutritional status and reducing toxicity from radiotherapy in CC patients (Aredes et al., 2019). However, the effect of pretreatment plasma omega-3 PUFAs levels on the clinical outcome of patients with CSCC who underwent CCRT has not been reported thus far. Therefore, predicting the survival rate by measuring the levels of omega-3 PUFAs before treatment in patients with CSCC who underwent CCRT may be helpful for evaluating patient prognosis.

The purpose of this experiment was to assess the predictive value of omega-3 PUFAs levels for early treatment response, progression-free survival (PFS), and overall survival (OS) in patients with CSCC who were undergoing CCRT.

## 2 Materials and methods

This study was approved by the Ethics Committee of the Second Affiliated Hospital of Dalian Medical University.

### 2.1 Study population

This study retrospectively included 89 patients with CSCC (stages IB3-IIIC2) who underwent CCRT at the Department of Radiotherapy Oncology, The Second Affiliated Hospital of Dalian Medical University from September 2019 to November 2021. The inclusion criteria of this study were as follows: (1) all patients were older than 30 years and were pathologically confirmed to have CSCC. Each patient was staged according to FIGO 2018. (2) Patients had no history of cancer or coexisting cancer. (3) Complete laboratory examination and clinical data, especially the serum SCC-Ag level before treatment and 1–3 months after CCRT. (4) Patients did not receive radiotherapy, chemotherapy, targeting, immunity and other treatments before collecting plasma omega-3PUFAs levels. Patients with the following characteristics were excluded from this study: (1) previous surgery for CC or cervical adenocarcinoma and adenosquamous carcinoma. (2) Patients who failed to complete CCRT for any reason. (3) Patients with any liver disease.

### 2.2 Clinical parameters and laboratory results

All the laboratory test and clinical data of the subjects, including age at diagnosis, BMI, tumor stage, maximum tumor diameter, lymph node metastasis, history of HPV infection, hypertension, history of diabetes and some routine laboratory examination data were obtained from the hospital electronic medical record database. Blood samples were collected on an empty stomach on the day of treatment, and 10 mL of blood was collected with an anticoagulant tube containing ethylenediaminetetraacetic acid (EDTA). The samples were centrifuged within 4 h to obtain plasma and a brownish yellow coating. Plasma samples were then collected and immediately stored at −80°C. The levels of EPA, DHA and DPA were detected via liquid chromatography-tandem mass spectrometry (LC-MS/MS) (Zhang et al., 2023). In our institution, the normal reference range for SCC-Ag is ≤ 2.5 ng/mL. In this study, patients with SCC-Ag>2.5 ng/mL, were divided into a group with high SCC-Ag levels. In contrast, when was SCC-Ag≤2.5 ng/mL, patients were classified into the low SCC-Ag subgroup.

### 2.3 CCRT treatment

All patients received external beam radiation therapy (EBRT) and brachytherapy. The EBRT dose was 45–50.4 Gy/25 to 28 fractions (1.8–2.0 Gy per fraction, 5 days per week). The irradiation area included the primary site, uterus, para-uterine region, and partial/full vaginal and pelvic lymph node metastasis/drainage areas. Brachytherapy was initiated near the end of EBRT as defined by the American Society for Brachytherapy guidelines. Brachytherapy was initiated at a dose of 6 Gy per fraction, two fractions per week, for a total dose of 24–30 Gy. Brachytherapy was not available on the same day as EBRT. CCRT was fully completed within 8 weeks. Chemotherapy consisted of weekly intravenous infusions of cisplatin or cisplatin combined with albumin-bound paclitaxel, administered every 3 weeks during radiotherapy.

### 2.4 Follow-up

The first follow-up examination was performed 1 month after the end of treatment. The data were reviewed every 3 months for the first 2 years after the completion of treatment, then every 6 months thereafter, and annually after 5 years. Routine follow-up included gynecological examinations, transvaginal color ultrasound, SCC-Ag measurements, enhanced chest CT and enhanced pelvic MRI, and enhanced upper and lower abdominal CT and so on. If recurrence is suspected in some patients, further pathologic biopsies and positron emission tomography-CT examinations will also be permitted. The primary endpoint was PFS, defined as the time from diagnosis of CC to local recurrence, distant metastasis, or last follow-up, and the secondary endpoint was OS, the time interval between diagnosis of CC and death from any cause or the date of last follow-up. Clinical response based on MRI scans was determined 3 months after the end of CCRT according to the RECIST 1.1 criteria (Schwartz et al., 2016). The response was divided into complete response (CR), partial response (PR), stable disease (SD) and progressive disease (PD). The patients were divided into two groups: the CR group and the non-CR group. The non-CR group included PR, PD and SD patients.

### 2.5 Statistical analysis

Student’s *t*-test was used to compare continuous variables with a normal distribution, expressed as the average ± standard deviation, while the Mann-Whitney U test was used for data with a nonnormal distribution, expressed as the range of the median (range). The chi-square test or Fisher’s exact test was used to compare classified variables in terms of quantities and percentages, and linear regression was used to check for potential collinearity between independent variables. Independent predictors associated with CR after CCRT were analyzed by including indicators that were meaningful as a single factor in a multifactorial binary logistic regression analysis. The OS and PFS curves were calculated by the Kaplan-Meier method with the log-rank test. Variables with *p* < 0.05 in univariate analysis were selected for multivariate Cox regression analysis to determine the independent predictors of survival outcome. Spearman rank correlation was used to analyze the correlation between significant FFAs and SCC-Ag. A *p*-value < 0.05 was considered to indicate statistical significance. The SPSS 26.0 software package was used for data processing.

## 3 Results

### 3.1 Study participant characteristics

The final study cohort consisted of 89 patients with CSCC, whose clinicopathologic characteristics are shown in Table 1, with a median follow-up of 41.3 months (range: 7.7–61.9 months). The median age at diagnosis of CC was 61 years (range: 30–81 years). The patients were staged according to the FIGO 2018 criteria, and the number of patients with stage IB3-II and III CC were 6 (6.7%), 38 (42.7%) and 45 (50.6%), respectively. In this study, lymph node metastasis was determined by a short diameter of lymph nodes ≥1 cm on MRI, of which 28 patients (31.5%) had lymph node metastasis and 61 patients (68.5%) were lymph node-negative. The median values of pre SCC-Ag and post SCC-Ag were 14.2 ng/mL (range: 5.0–52.2 ng/mL) and 1.7 ng/mL (range: 1.2–2.3 ng/mL) respectively. The plasma samples of 89 patients were evaluated by LC-MS/MS. The median level of omega-3 PUFAs in the whole cohort was as follows: EPA of 58.0 nmol/mL (range:42.5–86.0 nmol/mL); DHA of 301.0 nmol/mL (range: 223.0–364.0 nmol/mL), and DPA of 69.0 nmol/mL (range: 55.0–89.5 nmol/mL). MRI scans 3 months after the end of CCRT showed that 63 patients (70.8%) achieved CR and 26 patients (29.2%) did not achieve CR. The non-CR group included 24 (27.0%) patients with PR and 2 (2.2%) patients with PD or SD.

**TABLE 1:** The clinical characteristics of all patients (*n* = 89).

Factor	Total (N = 89)	%
Age (years)	61(53-68)	
BMI (kg/m^2^)		
<25	51	57.3
≥25	38	42.7
Hypertension history		
Yes	25	28.1
No	64	71.9
Diabetes history		
Yes	11	12.4
No	78	87.6
Gravidity		
0–2	48	53.9
≥3	28	31.5
Unknown	13	14.6
FIGO stage		
IB3	6	6.7
II	38	42.7
III	45	50.6
Size (cm)		
<4	26	29.2
≥ 4	63	70.8
Lymph nodes metastasis		
Positive	28	31.5
Negative	61	68.5
Pre SCC-Ag (ng/mL)	14.15(5.03-52.17)	
≤2.5	9	10.1
>2.5	80	89.9
Post SCC-Ag (ng/mL)	1.70(1.17-2.28)	
≤2.5	74	83.1
>2.5	15	16.9
HPV Status		
16+	35	39.3
Others	6	6.7
Negative	5	5.6
Unknown	43	48.3
HGB (g/L)	122.50±18.30	
EPA (nmol/mL)	58.00(42.50-86.00)	
DHA (nmol/mL)	301.00(223.00-364.00)	
DPA (nmol/mL)	69.00(55.00-89.50)	

Abbreviations: BMI, body mass index; HGB, hemoglobin; FIGO, international federation of gynecology and obstetrics; SCC-Ag, squamous cell carcinoma antigen; EPA, eicosapentaenoic acid; DHA, docosahexaenoic acid; DPA, docosapentaenoic acid.

### 3.2 Factors associated with CR after CCRT

CR after CCRT has been shown to be a reliable surrogate endpoint for survival in patients with LACC. Table 2 shows the relationship between the clinical response of CCRT and clinicopathologic characteristics, and univariate analysis showed that the response to CCRT was significantly correlated with the post SCC-Ag level (*p* = 0.010). The proportion of post SCC-Ag levels that were low (≤2.5 ng/mL) (90.5%) was much higher in the CCRT CR group than the proportion of post SCC-Ag levels that were high (>2.5 ng/mL) (9.5%). In the CCRT non-CR cohort, the proportion of patients with low levels of post SCC-Ag (≤2.5 ng/mL) (65.4%) was also greater than that of patients with high levels of post SCC-Ag (>2.5 ng/mL) (34.6%), but the difference was not as great as that in the effective group (Table 1). High levels of SCC-Ag after CCRT may be a risk factor for poor CCRT outcomes. Similarly, we also found that pretreatment plasma EPA levels were markedly higher in patients in the CCRT CR group than in the non-CR group, and that low levels of EPA were considerably linked with a reduced likelihood of CR (66.0 (48.0–90.0) nmol/mL vs 48.5 (31.8–69.3) nmol/mL, *p* = 0.018). DHA levels were slightly higher in the CR group than in the non-CR group, however, the results were not statistically different (*p* = 0.246). Similarly, the levels of DPA in the two groups were similar (*p* = 0.601). There were no differences between the two groups in term of BMI, age, tumor stage, incidence of positive lymph nodes, tumor size, and HPV type. We included factors significantly associated with response to CCRT in the univariate analysis via multifactorial binary logistic regression analysis to further identify factors influencing the prediction of CR after CCRT. Multivariate analysis revealed that post SCC-Ag levels >2.5 ng/mL (OR, 4.752; 95% CI, 1.431–15.786, *p* = 0.011) were independently associated with a reduced incidence of CR after CCRT. In contrast, high levels of plasma EPA were independently associated with an increased incidence of CR after CCRT (OR, 0.980; 95% CI, 0.962–0.999, *p* = 0.038), as shown in Table 3.

**TABLE 2:** Univariate analysis of clinical variables with response to CCRT.

	CR (n=63)	Non-CR (n=26)	*P*
Age (years)	61(53-69)	60(52-66)	0.838
<60	30(47.6)	13(50.0)	
≥60	33(52.4)	13(50.0)	
BMI (kg/m^2^)			
<25	34(54)	17(65.4)	0.322
≥25	29(46)	9(34.6)	
Hypertension history			
Yes	19(30.2)	6(23.1)	0.499
No	44(69.8)	20(76.9)	
Diabetes history			
Yes	5(7.9)	6(23.1)	0.105
No	58(92.1)	20(76.9)	
Gravidity			
0–2	35(55.6)	13(50)	0.636
≥3	18(28.6)	10(38.5)	
Unknown	10(15.9)	3(11.5)	
FIGO stage			
IB3	6(9.5)	0(0)	0.173
II	28(44.4)	10(38.5)	
III	29(46.0)	16(61.5)	
Size (cm)			
<4	19(20.2)	7(26.9)	0.760
≥ 4	44(69.8)	19(73.1)	
Lymph nodes metastasis			
Positive	17(27.0)	11(42.3)	0.157
Negative	46(73.0)	15(57.7)	
Pre SCC-Ag (ng/mL)	12.95(6.93-40.76)	16.58(4.38-54.46)	
≤2.5	6(9.5)	3(11.5)	1.000
>2.5	57(90.5)	23(88.5)	
Post SCC-Ag (ng/mL)	1.60(1.16-2.05)	2.00(1.24-2.80)	
≤2.5	57(90.5)	17(65.4)	0.010
>2.5	6(9.5)	9(34.6)	
HPV Status			
16+	26(41.3)	9(34.6)	0.871
Others	4(6.3)	2(7.7)	
Negative	4(6.3)	1(3.8)	
Unknown	29(46)	14(53.8)	
HGB (g/L)	123.80±17.40	119.50±20.50	0.319
EPA (nmol/mL)	66.00 (48.00-90.00)	48.50 (31.75-69.25)	0.018
DHA (nmol/mL)	303.00 (246.00-375.00)	289.00 (192.80-354.50)	0.246
DPA (nmol/mL)	69.00 (55.00-91.00)	71.00 (53.30-89.25)	0.601

Abbreviations: BMI, body mass index; HGB, hemoglobin; FIGO, international federation of gynecology and obstetrics; SCC-Ag, squamous cell carcinoma antigen; CR, complete response; EPA, eicosapentaenoic acid; DHA, docosahexaenoic acid; DPA, docosapentaenoic acid.

**TABLE 3:** Multivariate analysis of clinical variables with response to CCRT.

	β	OR (95% CI)	*P*
Post SCC-Ag	1.559	4.752 (1.431-15.786)	0.011
EPA	-0.020	0.980 (0.962-0.999)	0.038

Abbreviations: SCC-Ag, squamous cell carcinoma antigen; OR, odds ratio; 95% CI, 95% confidence interval; EPA, eicosapentaenoic acid.

### 3.3 Optimal cut-off value for omega −3 PUFAs in plasma before CCRT

Optimal AUC and cut-off value for plasma omega-3 PUFAs parameters prior to CCRT in patients with CSSS were determined by plotting ROC curves for survival outcomes. The cut-off value for the joint maximum sensitivity and specificity of EPA was 46.0 nmol/mL (*p* = 0.029, AUC = 0.713, 95% CI = 0.561–0.865), that of DHA was 308.0 nmol/mL (*p* = 0.036, AUC = 0.704, 95% CI = 0.549–0.858) and that of DPA was 69.0 nmol/mL (*p* = 0.740, AUC = 0.532, 95% CI = 0.363–0.702). For further analysis, patients were categorized into high EPA or low EPA group (≥46.0 nmol/mL or <46.0 nmol/mL, respectively), high DHA or low DHA group (≥308.0 nmol/mL or <308.0 nmol/mL, respectively) and high DPA or low DPA group (≥69.0 nmol/mL or <69.0 nmol/mL, respectively).

### 3.4 Survival analysis

**FIGURE 1:**
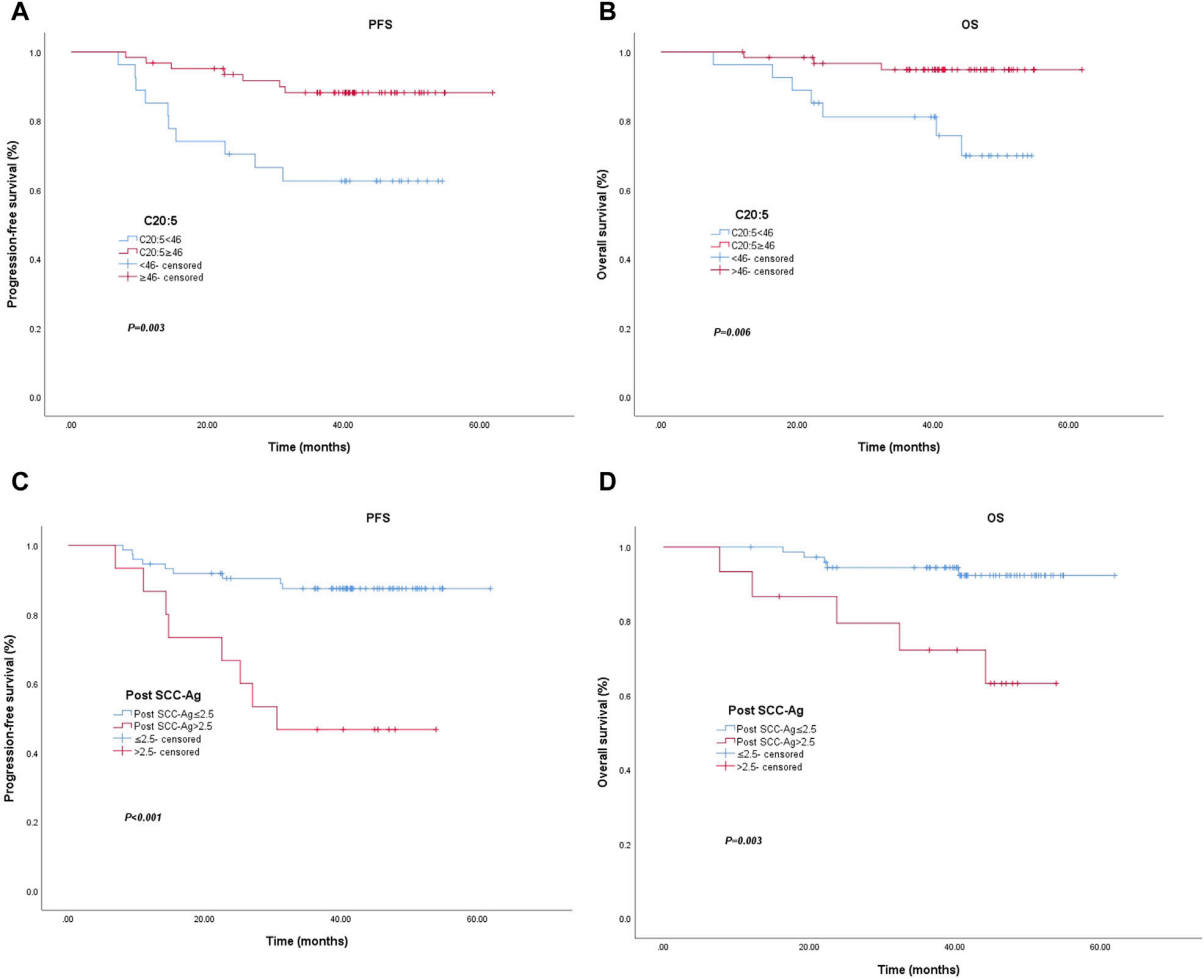
Kaplan–Meier curve for progression-free survival and overall survival for patients with low vs. high C20:5 levels **(A)** (*p* = 0.003) and **(B)** (*p* = 0.006). Kaplan–Meier curve for progression-free survival and overall survival patients with low vs. high post SCC-Ag levels **(C)** (*p* < 0.001) and **(D)** (*p* = 0.003). Abbreviations: Post SCC-Ag, posttreatment squamous cell carcinoma antigen; C20:5, eicosapentaenoic acid, EPA.

Of the 89 patients, 10 (37.0%) of 27 patients with an EPA level <46.0 nmol/mL were diagnosed with locally recurrent or metastatic disease, and 7 (11.3%) of 62 patients with an EPA level ≥46.0 nmol/mL were diagnosed with locally recurrent or metastatic disease (*p* = 0.003). The PFS rates of the low EPA group at 1 and 3 years were 85.2% and 62.6%, respectively, and the PFS rates of the high EPA group at 1 and 3 years were 96.8% and 88.1%, respectively. The PFS data are shown in Figure 1A. Patients with EPA levels ≥46.0 nmol/mL had significantly better PFS than patients with EPA levels <46.0 nmol/mL. In terms of OS, 7 (25.9%) of 27 patients with EPA levels <46.0 nmol/mL died, and 3 (4.8%) of 62 patients with EPA levels ≥46.0 nmol/mL died (*p* = 0.006). OS was 96.3% and 81.1% at 1 and 3 years in the low EPA group and 98.4% and 94.8% at 1 and 3 years in the high EPA group, respectively. The OS data are shown in Figure 1B. Patients with EPA levels <46.0 nmol/mL had relatively poor OS. Similarly, the differences in 3-year PFS and OS between patients with post SCC-Ag levels ≤2.5 ng/mL and those with >2.5 ng/mL were statistically significant in Figure 1C,D.

Table 4 and Table 5 summarize the univariate and multivariate HR and 95% CI for PFS and OS (HPV status was not included in the survival analysis because of missing data). Univariate analysis revealed that pretreatment plasma EPA ≥46.0 nmol/mL levels and DHA≥308.0 nmol/mL levels were considerably connected with improvement in PFS (HR, 0.259; 95% CI, 0.099–0.682, *p* = 0.006) and (HR, 0.309; 95% CI, 0.101–0.949, *p* = 0.040). Other clinicopathologic variables that were dramatically linked with improvement in PFS included achievement of CR after CCRT (HR, 0.238; 95% CI, 0.090–0.625, *p* = 0.004). Lymph node metastasis, later FIGO staging, and post SCC-Ag level >2.5 ng/mL were significantly linked with decreased PFS (all *p* < 0.05). In the Multivariate analysis, pretreatment plasma EPA level ≥46.0 nmol/mL remained an independent prognostic factor for improved PFS (HR, 0.263; 95% CI, 0.089–0.782, *p* = 0.016). Other independent factors associated with reduced PFS included lymph node metastasis (HR, 4.678; 95% CI, 1.576–13.886, *p* = 0.005), and post SCC-Ag level >2.5 ng/mL (HR, 3.148; 95% CI, 1.138–8.706, *p* = 0.027).

**TABLE 4:** Univariate and multivariate analyses of prognostic factors for PFS among patients with CC.

	Univariate		Multivariate	
Characteristics	HR (95% CI)	*P*	HR (95% CI)	*P*
Age (years)				
(<60 vs. ≥60)	0.829(0.319-2.145)	0.697		
BMI (kg/m^2^)				
(≥25 vs. <25)	0.525(0.185-1.490)	0.226		
Hypertension history				
(yes vs. no)	0.520(0.149-1.810)	0.520		
Diabetes history				
(yes vs. no)	0.391(0.052-2.949)	0.362		
Lymph nodes metastasis				
(yes vs. no)	5.378(1.982-14.594)	0.001	4.678(1.576-13.886)	0.005
FIGO stage				
IB3	3.508(1.213-10.144)	0.021	1.992(0.604-6.568)	0.257
II				
III				
Pre SCC-Ag (ng/mL)				
(>2.5 vs.≤2.5)	1.957(0.259-14.760)	0.515		
Post SCC-Ag (ng/mL)				
(>2.5 vs.≤2.5)	5.296(2.036-13.776)	0.001	3.148(1.138-8.706)	0.027
Size (cm)				
(<4 vs. ≥4)	0.989(0.348-2.808)	0.984		
CR achieved				
(yes vs. no)	0.238(0.090-0.625)	0.004	0.466(0.167-1.306)	0.146
HGB (g/L)				
(≥110 vs.<110)	0.631(0.222-1.794)	0.388		
EPA (nmol/mL)				
(≥46 vs.<46)	0.259(0.099-0.682)	0.006	0.263(0.089-0.782)	0.016
DPA (nmol/mL)				
(≥69 vs.<69)	0.463(0.171-1.254)	0.130		
DHA (nmol/mL)				
(≥308 vs.<308)	0.309(0.101-0.949)	0.040	0.525(0.157-1.759)	0.296

Abbreviations: HR, hazard ratio; 95% CI, 95% confidence interval; BMI, body mass index; HGB, hemoglobin; FIGO, international federation of gynecology and obstetrics; SCC-Ag, squamous cell carcinoma antigen; EPA, eicosapentaenoic acid; DHA, docosahexaenoic acid; DPA, docosapentaenoic acid.

**TABLE 5:** Univariate and multivariate analyses of prognostic factors for OS among patients with CC.

	Univariate		Multivariate	
Characteristics	HR (95% CI)	*P*	HR (95% CI)	*P*
Age (years)				
(<60 vs. ≥60)	1.428(0.402-5.073)	0.581		
BMI (kg/m^2^)				
(≥25 vs. <25)	0.339(0.072-1.597)	0.171		
Hypertension history				
(yes vs. no)	0.262(0.033-2.070)	0.204		
Diabetes history				
(yes vs. no)	0.792(0.100-6.285)	0.825		
Lymph nodes metastasis				
(yes vs. no)	6.951(1.787-27.040)	0.005	7.409(1.760-31.194)	0.006
FIGO stage				
IB3	3.957(0.898-17.439)	0.069		
II				
III				
Post SCC-Ag (ng/mL)				
(>2.5 vs.≤2.5)	5.307(1.530-18.405)	0.009	2.916(0.802-10.605)	0.104
Size (cm)				
(<4 vs. ≥4)	0.634(0.179-2.248)	0.480		
CR achieved				
(yes vs. no)	0.225(0.063-0.800)	0.021	0.232(0.060-0.896)	0.034
HGB (g/L)				
(≥110 vs.<110)	0.604(0.156-2.341)	0.466		
EPA (nmol/mL)				
(≥46 vs.<46)	0.182(0.047-0.705)	0.014	0.288(0.063-1.313)	0.108
DPA (nmol/mL)				
(≥69 vs.<69)	0.521(0.147-1.856)	0.315		
DHA (nmol/mL)				
(≥308 vs.<308)	0.109(0.014-0.862)	0.036	0.146(0.015-1.391)	0.094

Abbreviations: HR, hazard ratio; 95% CI, 95% confidence interval; BMI, body mass index; HGB, hemoglobin; FIGO, international federation of gynecology and obstetrics; SCC-Ag, squamous cell carcinoma antigen; EPA, eicosapentaenoic acid; DHA, docosahexaenoic acid; DPA, docosapentaenoic acid.

Univariate analysis showed that achieving CR after CCRT was significantly associated with improved OS (HR, 0.225; 95% CI, 0.063–0.800, *p* = 0.021). Pretreatment plasma EPA ≥46.0 nmol/mL level and DHA≥308.0 nmol/mL level were considerably connected with improvement in OS (HR, 0.182; 95% CI, 0.047–0.705, *p* = 0.014) and (HR, 0.109; 95% CI, 0.014–0.862, *p* = 0.036). Similarly, we also observed that lymph node metastasis (HR, 6.951; 95% CI, 1.787–27.040, *p* = 0.005), and post SCC-Ag level >2.5 ng/mL (HR, 5.307; 95% CI, 1.530–18.405, *p* = 0.009) were significantly associated with decreased OS. Through multivariate analysis, only lymph node metastasis was the independent predictor of OS reduction (HR, 7.409; 95% CI, 1.760–31.194, *p* = 0.006). CR after CCRT was an independent prognostic factor for improved OS (HR, 0.232; 95% CI, 0.060–0.896, *p* = 0.034.). Multivariate analysis showed that pretreatment EPA ≥46.0 nmol/mL level was not an independent predictor of OS (HR, 0.288; 95% CI, 0.063–1.313, *p* = 0.108). Similarly, pretreatment DHA ≥308.0 nmol/mL level was not an independent predictor of OS (HR, 0.146; 95% CI, 0.015–1.391, *p* = 0.094).

### 3.5 Relationship between the EPA level and other clinical characteristics

The clinicopathological features of the two groups were compared, as shown in Table 6. The statistical analysis showed that EPA level was significantly correlated with age (*p* = 0.022), BMI (*p* = 0.002), and CR after CCRT (*p* = 0.037). However, there was no significant relationship between EPA level and other parameters.

**TABLE 6:** Relationships between clinicopathological data and the different levels of pretreatment C20:5 in CC.

	C20:5<46	C20:5≥46	
Characteristics	N=27	N=62	*P*
Age (years)			
<60	18(66.7)	25(40.3)	0.022
≥60	9(33.3)	37(59.7)	
BMI (kg/m^2^)			
<25	22(81.5)	29(46.8)	0.002
≥25	5(18.5)	33(53.2)	
Hypertension history			
yes	6(22.2)	19(30.6)	0.416
no	21(77.8)	43(69.4)	
Diabetes history			
yes	1(3.7)	10(16.1)	0.198
no	26(96.3)	52(83.9)	
Lymph nodes metastasis			
yes	8(29.6)	20(32.3)	0.806
no	19(70.4)	42(67.7)	
FIGO stage			
IB3	1(3.7)	5(8.1)	0.483
II	10(37.0)	28(45.2)	
III	16(59.3)	29(46.8)	
Pre SCC-Ag (ng/mL)			
≤2.5	1(3.7)	8(12.9)	0.347
>2.5	26(96.3)	54(87.1)	
Post SCC-Ag (ng/mL)			
≤2.5	21(77.8)	53(85.5)	0.559
>2.5	6(22.2)	9(14.5)	
Size(cm)			
<4	7(25.9)	19(30.6)	0.653
≥4	20(74.1)	43(69.4)	
CR achieved			
yes	15(55.6)	48(77.4)	0.037
no	12(44.4)	14(22.6)	
HGB (g/L)			
<110	8(29.6)	12(19.4)	0.286
≥110	19(70.4)	50(80.6)	

Abbreviations: BMI, body mass index; HGB, hemoglobin; FIGO, international federation of gynecology and obstetrics; SCC-Ag, squamous cell carcinoma antigen**;** C20:5, eicosapentaenoic acid, EPA.

### 3.6 Correlation of SCC-Ag levels with plasma EPA levels

**FIGURE 2:**
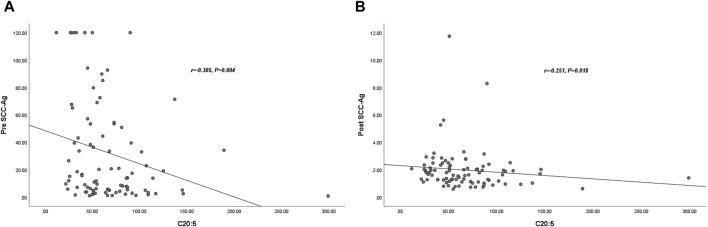
Correlation between plasma C20:5 and Post- SCC-Ag levels in 89 patients with CC. The plasma C20:5 levels were negatively correlated with pre SCC-Ag (*r* = −0.305, *p* = 0.004) **(A)**. The plasma C20:5 levels were negatively correlated with post SCC-Ag levels (*r* = −0.251, *p* = 0.018) **(B)**. Abbreviations: SCC-Ag, squamous cell carcinoma antigen; C20:5, eicosapentaenoic acid, EPA; Pre SCC-Ag, pretreatment squamous cell carcinoma antigen; Post SCC-Ag, posttreatment squamous cell carcinoma antigen.

To confirm the relationship between SCC-Ag levels and plasma EPA levels, we performed Spearman rank correlation analysis. The results showed that pre SCC-Ag and EPA were negatively correlated (*r* = −0.305, *p* = 0.004), and post SCC-Ag and EPA were weakly negatively correlated (*r* = −0.251, *p* = 0.018), as shown in Figures 2A,B.

### 3.7 Prognostic significance of combining plasma EPA and post-SCC ag

**FIGURE 3:**
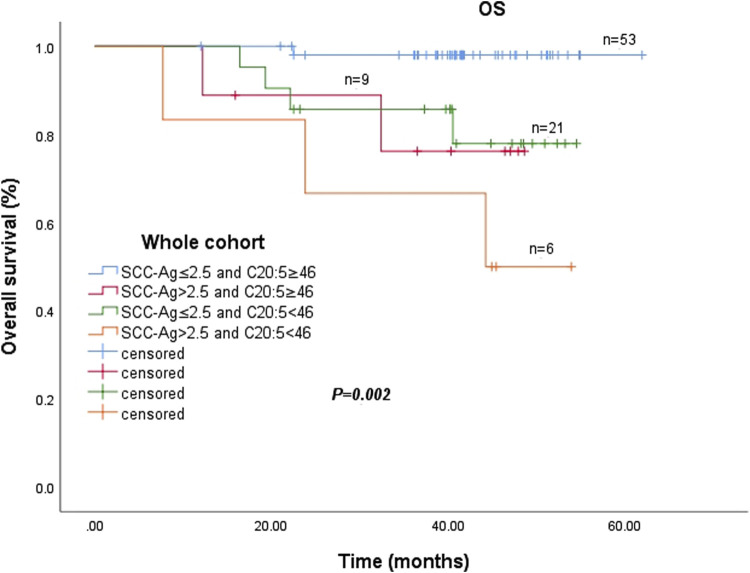
Abbreviations: SCC-Ag, squamous cell carcinoma antigen; C20:5, eicosapentaenoic acid, EPA.

The prognostic significance of combining EPA and post SCC-Ag levels in the entire cohort is shown in Figure 3. There was a significant difference in the 3-year OS rate among the four groups (*p* = 0.002). The pretreatment plasma EPA levels ≥46.0 nmol/mL and post SCC-Ag levels ≤2.5 ng/mL groups had significantly greater survival probabilities than the other three groups. In contrast, patients with pretreatment plasma EPA levels <46.0 nmol/mL and post SCC-Ag levels >2.5 ng/mL had the lowest survival rate among the four groups in Figure 3.

## 4 Discussion

In view of the poor prognosis of patients with CC and the high tumor recurrence rate after CCRT, it is necessary to screen the risk factors of tumor prognosis and recurrence in order to support early intervention, improve monitoring, reduce tumor recurrence rate and prolong patient survival time. As lipids are closely related to tumorigenesis or tumor recurrence, marine polyunsaturated fatty acids (omega-3 PUFAs) as an important lipid, it is necessary to understand whether there is a relationship between omega-3PUFAs and the response and prognosis of patients with CC who underwent CCRT. This is the first study to evaluate the association between omega-3 PUFAs and response and prognosis in patients with CSCC who received CCRT.

Previous *in vivo* and *in vitro* studies have found that in addition to its direct cytotoxic effects on tumor cells, EPA can increase the cytotoxicity of anticancer drugs to tumors or cancer cells, even at doses that have no substantial effect on tumor growth or cell viability (Bégin et al., 1986; Bégin et al., 1988). Early experiments have proven that omega-3 PUFAs represented by EPA can enhance the sensitivity of breast, colorectal and ovarian cancers and other tumors to anticancer drugs, and have cytotoxicity to both vincristine-sensitive and drug-resistant human CC cells, which can increase the killing effect of human CC cells by 30%, and increase cisplatin’s cytotoxicity against cytotoxicity of cisplatin on human CC cells by 36%–60% (Das et al., 1998; Hajjaji and Bougnoux, 2013). Consistent with these findings, our study found that both univariate and multivariate analysis results showed that patients who achieved CR in patients with CSCC who underwent CCRT had higher pretreatment levels of EPA. This finding suggested that a high level of EPA before treatment is an independent predictor of CR after CCRT. We suspect the mechanism might be EPA is the main starting substance of lipid peroxidation. With the increase in EPA concentration, the level of lipid peroxide produced by cells increased significantly, which was related to the loss of cell vitality and the significant inhibition of cell growth (Falconer et al., 1994). EPA treatment promotes the production of large amounts of ROS, which are highly peroxidizable and therefore may enhance the efficacy of cytotoxic ROS-inducing drugs against tumors (such as anthracycline, mitomycin, etc.) (Das et al., 1998; Colquhoun, 2009; Moon, 2023). Since ionizing radiation also generates ROS, which leads to lipid peroxidation of PUFAs, lipid peroxidation may be further enhanced in the membranes of EPA-rich tumor cells, and thus the combination of EPA and radiotherapy may cause additional damage to tumor cells (Manda et al., 2011; Zaloga, 2021). This seems to provide a strong explanation for the fact that EPA can enhance the sensitivity of radiotherapy and chemotherapy.

Regarding the relationship between EPA and tumor prognosis has rarely been investigated, a phase II randomized, double-blind trial demonstrated that oral EPA significantly slowed the growth of colorectal liver metastases, the reason for which may be that EPA has antiangiogenic properties and limited preoperative EPA exposure may prolong disease-free survival (DFS) and OS (Cockbain et al., 2014). Murphy et al. also demonstrated that NSCLC patients treated with platinum-based chemotherapy taking 2.5 g of EPA + DHA supplementation per day had a twofold increase in clinical benefit and treatment response rate, and a higher 1-year survival rate for patients in the supplementation group (60% vs 38.7%), than patients who received standard treatment but no additional supplementation (Murphy et al., 2011). These studies are consistent with our results. We found that in patients with CSCC treated with CCRT, the level of plasma EPA ≥46.0 nmol/mL before treatment was significantly correlated with PFS (HR, 0.263; 95% CI, 0.089–0.782, *p* = 0.016). In multivariate Cox regression analysis, it was found that plasma EPA ≥46.0 nmol/mL before treatment was not an independent predictor of OS (HR, 0.288; 95% CI, 0.063–1.313, *p* = 0.108). However, the Kaplan-Meier survival curve showed that the OS of patients with plasma EPA ≥46.0 nmol/mL was better than that of patients with plasma EPA <46.0 nmol/mL before treatment. The reason for this phenomenon may be due to the small sample size and insufficient follow-up time. We will strive to collect more samples and conduct long-term follow-up for further research. However, in the univariate analysis of PFS and OS, the plasma DHA level ≥308.0 nmol/mL before treatment was statistically significant, *p* = 0.040 and 0.036, respectively, but in multivariate analysis, DHA level was not an independent prognostic factor for PFS and OS.

This study suggested that plasma EPA is a valid pretreatment biomarker for predicting survival in CSCC patients who underwent CCRT. However, the possible mechanism by which pretreatment plasma EPA reflects the prognosis of patients with CC as a new indicator for assessing OS and PFS is unclear. The preliminary explanation can only be given by considering previous studies. The anticancer effects of EPA are mainly attributed to its ability to modulate cell death pathways, inhibit cell proliferation and induce the expression of anti-inflammatory mediators (D'Angelo et al., 2020; Montecillo-Aguado et al., 2023). The mechanism by which EPA promotes apoptosis alone involves the alteration of multiple complex cellular pathways; for example, EPA was able to induce cell cycle arrest in breast cancer cell lines (Zhu et al., 2018). In particular, the progression of the cell cycle from the S to the G2-M phase was blocked in BT20 breast cancer cells, and this apoptotic effect was shown in a concentration- and time-dependent manner (Nappo et al., 2012). Similarly, it inhibits cholesterol efflux channel protein (ABCA1), leading to intracellular cholesterol accumulation and thus dysregulation of cholesterol homeostasis, which in turn increases cell membrane polarity lethality in triple-negative breast cancer cells (Torres-Adorno et al., 2019).

As a tumor marker, SCC-Ag is widely used in CSCC, and the increase of SCC-Ag before treatment is associated with larger tumor size, late tumor stage, regional lymph node involvement and deep stroma involvement, so it is widely used in monitoring CC recurrence (Wang et al., 2019). However, there are few studies on the relationship between SCC-Ag and the prognosis of CC. Wang et al. included 559 patients with CSCC. Multivariate analysis showed that SCC-Ag did not decrease to normal after CCRT was an independent prognostic factor for DFS (HR, 5.10; 95%CI, 3.31–7.88, *p* < 0.001) (Wang et al., 2019). This is highly consistent with our results, which show that post SCC-Ag level>2.5 ng/mL is an independent risk factor for 3-year PFS (HR, 3.148; 95%CI, 1.138–8.706, *p* = 0.027). Interestingly, we found that there was a negative correlation between pre SCC-Ag and EPA (*r* = −0.305, *p* = 0.004),a weak negative correlation between post SCC-Ag and EPA (*r* = −0.251, *p* = 0.018) and the survival probability of patients in the pretreatment plasma EPA≥46.0 nmol/mL and post SCC-Ag≤2.5 ng/mL groups had the highest probability of survival, and patients with pretreatment plasma EPA<46.0 nmol/mL and post SCC-Ag>2.5 ng/mL had the lowest survival rate among the four groups. These results further confirmed the inverse relationship between EPA and SCC-Ag. Recent studies have also shown that EPA can significantly reduce the levels of serum TNF- α and IL-6 in tumor patients. At the same time, EPA can reduce the level of inflammatory reaction and improve body immune function by reducing the production of C-reactive protein and limiting the release of IL-6 (Liu et al., 2023). It is suggested that the expression levels of EPA and post SCC-Ag may be auxiliary markers for monitoring disease progression and tumor malignancy. However, we found that FIGO staging was a significant predictor of PFS in univariate analysis, but its significance was not significant in multivariate analysis. One of the reasons for this phenomenon may be the insufficient number of cases studied, and the lack of stage IVA patients in our group may also be.

EPA has a wide range of molecular targets in cancer cells and tumors. To sum up, the results show that EPA can activate a variety of molecular biological processes, including classical and alternative apoptosis pathways, and regulate survival and cell growth signals. Causing cell death in a variety of cells and animal models. However, further experiments are needed to explore the specific details of external approaches, especially upstream events. These findings suggest that EPA is expected to be a potential new treatment for cancer and can complement existing treatments. However, our clinical study further validates these basic experiments. In conclusion, the above mechanisms may partially explain why pretreatment plasma EPA may be a biomarker of outcome and prognosis in patients with CSCC who underwent CCRT. This study showed that the abnormal level of plasma EPA before treatment is directly related to the response and survival outcome of patients with CSCC receiving CCRT, and the level of plasma EPA before treatment can be used as a biomarker to assess the response and prognosis of CSCC patients.

However, our study also has some limitations. First, as a small-sample retrospective study, we regret that we were unable to accurately and dynamically track the level of changes in omega-3 PUFAs during and at the end of CCRT. Second, this study did not examine the relationship between nutritional support therapy and omega-3 PUFAs levels. Finally, the patients we enrolled were all patients with CSCC, and the results may be biased when it comes to patients with types of cervical adenocarcinoma and adenosquamous carcinoma. In the future, it is necessary to further elucidate the specific molecular mechanisms of the regulation of apoptosis, proliferation, survival, migration and drug resistance of omega-3 PUFAs, including the specific pathways involved and the interactions between different PUFAs. In addition, more research is needed to investigate the effects of omega-3 PUFAs on different types of cancer cells and animal models, and to evaluate their potential synergies with existing cancer therapies. However, it is unclear whether there is a clear benefit of elevated EPA levels after supplemental therapy, and if their beneficial effect is confirmed to improve local control of cervical cancer through dietary lipids as an adjunctive to CCRT, the prospects for systemic supplementation during CSCC treatment would be considerable.

## 5 Conclusion

In conclusion, the present study demonstrated that pretreatment plasma omega-3 PUFAs are associated with survival outcomes in patients with CSCC treated with CCRT. Pretreatment plasma EPA may be a promising biomarker for predicting recent response and PFS in patients with CSCC who underwent CCRT. The use of plasma EPA level before treatment and serum SCC-Ag level after treatment increased the prognostic significance of serum SCC-Ag level alone. Perhaps it is preferable to use a system of two or more determinants to create new prognostic tools for clinicians in CSCC. As an independent factor, EPA can be used as a new and promising prognostic index, which can be detected routinely and conveniently and cheaply, which will provide potential prognostic information for clinicians to promote individualized treatment of patients with CSCC.

## Data Availability

The original contributions presented in the study are included in the article/Supplementary Material, further inquiries can be directed to the corresponding authors.
