# Supplementary material for: The value of plasma omega-3 polyunsaturated fatty acids in predicting the response and prognosis of cervical squamous cell carcinoma patients to concurrent chemoradiotherapy
Source: Front Pharmacol. 2024 May 27;15:1379508. doi: 10.3389/fphar.2024.1379508 (PMC11163051; doi:10.3389/fphar.2024.1379508)
Supplement: Supplementary file 1 [file DataSheet1.PDF]

Supplementary Material

Supplementary Tables

Table 1. The clinical characteristics of all patients (n = 89)

Factor	Total (N = 89)	%
Age (years)	61(53-68)	
BMI (kg/m^2)		
<25	51	57.3
≥25	38	42.7
Hypertension history		
Yes	25	28.1
No	64	71.9
Diabetes history		
Yes	11	12.4
No	78	87.6
Gravidity		
0–2	48	53.9
⩾3	28	31.5
Unknown	13	14.6
FIGO stage		
IB3	6	6.7
II	38	42.7
III	45	50.6
Size (cm)		
＜4	26	29.2
≥ 4	63	70.8
Lymph nodes metastasis		
Positive	28	31.5
Negative	61	68.5
Pre SCC-Ag (ng/mL)	14.15(5.03-52.17)	
⩽2.5	9	10.1
>2.5	80	89.9
Post SCC-Ag (ng/mL)	1.70(1.17-2.28)	
⩽2.5	74	83.1
>2.5	15	16.9
HPV Status		
16+	35	39.3
Others	6	6.7
Negative	5	5.6
Unknown	43	48.3
HGB (g/L)	122.50±18.30	
EPA (nmol/mL)	58.00(42.50-86.00)	
DHA (nmol/mL)	301.00(223.00-364.00)	
DPA (nmol/mL)	69.00(55.00-89.50)	

Table 1. Abbreviations: BMI, body mass index; HGB, hemoglobin; FIGO, International Federation of Gynecology and Obstetrics; SCC-Ag, squamous cell carcinoma antigen; EPA, Eicosapentaenoic acid; DHA, Docosahexaenoic acid; DPA, Docosapentaenoic acid.

6 **Table 2. Univariate analysis of clinical variables with response to CCRT.**

Table 2. Abbreviations: BMI, body mass index; HGB, hemoglobin; FIGO, International Federation

	CR (n=63)	Non-CR (n=26)	*P*
Age (years)	61(53-69)	60(52-66)	
<60	30(47.6)	13(50.0)	0.838
≥60	33(52.4)	13(50.0)	
BMI (kg/m^2)			
<25	34(54)	17(65.4)	0.322
≥25	29(46)	9(34.6)	
Hypertension history			
Yes	19(30.2)	6(23.1)	0.499
No	44(69.8)	20(76.9)	
Diabetes history			
Yes	5(7.9)	6(23.1)	0.105
No	58(92.1)	20(76.9)	
Gravidity			
0–2	35(55.6)	13(50)	0.636
⩾3	18(28.6)	10(38.5)	
Unknown	10(15.9)	3(11.5)	
FIGO stage			
IB3	6(9.5)	0(0)	0.173
II	28(44.4)	10(38.5)	
III	29(46.0)	16(61.5)	
Size (cm)			
＜4	19(20.2)	7(26.9)	0.760
≥ 4	44(69.8)	19(73.1)	
Lymph nodes metastasis			
Positive	17(27.0)	11(42.3)	0.157
Negative	46(73.0)	15(57.7)	
Pre SCC-Ag (ng/mL)	12.95(6.93-40.76)	16.58(4.38-54.46)	
⩽2.5	6(9.5)	3(11.5)	1.000
>2.5	57(90.5)	23(88.5)	
Post SCC-Ag (ng/mL)	1.60(1.16-2.05)	2.00(1.24-2.80)	
⩽2.5	57(90.5)	17(65.4)	0.010
>2.5	6(9.5)	9(34.6)	
HPV Status			
16+	26(41.3)	9(34.6)	0.871
Others	4(6.3)	2(7.7)	
Negative	4(6.3)	1(3.8)	
Unknown	29(46)	14(53.8)	
HGB (g/L)	123.80±17.40	119.50±20.50	0.319
EPA (nmol/mL)	66.00 (48.00-90.00)	48.50 (31.75-69.25)	0.018
DHA (nmol/mL)	303.00 (246.00-375.00)	289.00 (192.80-354.50)	0.246
DPA (nmol/mL)	69.00 (55.00-91.00)	71.00 (53.30-89.25)	0.601

of Gynecology and Obstetrics; SCC-Ag, squamous cell carcinoma antigen; CR, complete response; EPA, Eicosapentaenoic acid; DHA, Docosahexaenoic acid; DPA, Docosapentaenoic acid.

Table 3. Multivariate analysis of clinical variables with response to CCRT.

	β	OR (95% CI)	*P*
Post SCC-Ag	1.559	4.752 (1.431-15.786)	0.011
EPA	-0.020	0.980 (0.962-0.999)	0.038

Table 3. Abbreviations: SCC-Ag, squamous cell carcinoma antigen; OR, odds ratio; 95% CI, 95% confidence interval; EPA, Eicosapentaenoic acid.

Table 4. Univariate and multivariate analyses of prognostic factors for PFS among patients with CC

	Univariate	Multivariate

Characteristics	HR (95% CI)	*P*	HR (95% CI)	*P*
Age (years)				
(<60 vs. ≥60)	0.829(0.319-2.145)	0.697		
BMI (kg/m^2)				
(≥25 vs. <25)	0.525(0.185-1.490)	0.226		
Hypertension history				
(yes vs. no)	0.520(0.149-1.810)	0.520		
Diabetes history				
(yes vs. no)	0.391(0.052-2.949)	0.362		
Lymph nodes metastasis				
(yes vs. no)	5.378(1.982-14.594)	0.001	4.678(1.576-13.886)	0.005
FIGO stage				
IB3	3.508(1.213-10.144)	0.021	1.992(0.604-6.568)	0.257
II				
III				
Pre SCC-Ag (ng/mL)				
(>2.5 vs.≤2.5)	1.957(0.259-14.760)	0.515		
Post SCC-Ag (ng/mL)				
(>2.5 vs.≤2.5)	5.296(2.036-13.776)	0.001	3.148(1.138-8.706)	0.027
Size (cm)				
(<4 vs. ≥4)	0.989(0.348-2.808)	0.984		
CR achieved				
(yes vs. no)	0.238(0.090-0.625)	0.004	0.466(0.167-1.306)	0.146
HGB (g/L)				
(≥110 vs.<110)	0.631(0.222-1.794)	0.388		
EPA (nmol/mL)				
(≥46 vs.<46)	0.259(0.099-0.682)	0.006	0.263(0.089-0.782)	0.016
DPA (nmol/mL)				
(≥69 vs.<69)	0.463(0.171-1.254)	0.130		
DHA (nmol/mL)				
(≥308 vs.<308)	0.309(0.101-0.949)	0.040	0.525(0.157-1.759)	0.296

Table 4. Abbreviations: HR, hazard ratio; 95% CI, 95% confidence interval; BMI, body mass index; HGB, hemoglobin; FIGO, International Federation of Gynecology and Obstetrics; SCC-Ag, squamous cell carcinoma antigen; EPA, Eicosapentaenoic acid; DHA, Docosahexaenoic acid; DPA, Docosapentaenoic acid.

Table 5. Univariate and multivariate analyses of prognostic factors for OS among patients with CC

	Univariate		Multivariate	
**Characteristics**	**HR (95% CI)**	***P***	**HR (95% CI)**	***P***
Age (years)				
(<60 vs. ≥60)	1.428(0.402-5.073)	0.581		
BMI (kg/m^2)				
(≥25 vs. <25)	0.339(0.072-1.597)	0.171		
Hypertension history				
(yes vs. no)	0.262(0.033-2.070)	0.204		
Diabetes history				

(yes vs. no)	0.792(0.100-6.285)	0.825		
Lymph nodes metastasis				
(yes vs. no)	6.951(1.787-27.040)	0.005	7.409(1.760-31.194)	0.006
FIGO stage				
IB3	3.957(0.898-17.439)	0.069		
II				
III				
Post SCC-Ag (ng/mL)				
(>2.5 vs.≤2.5)	5.307(1.530-18.405)	0.009	2.916(0.802-10.605)	0.104
Size (cm)				
(<4 vs. ≥4)	0.634(0.179-2.248)	0.480		
CR achieved				
(yes vs. no)	0.225(0.063-0.800)	0.021	0.232(0.060-0.896)	0.034
HGB (g/L)				
(≥110 vs.<110)	0.604(0.156-2.341)	0.466		
EPA (nmol/mL)				
(≥46 vs.<46)	0.182(0.047-0.705)	0.014	0.288(0.063-1.313)	0.108
DPA (nmol/mL)				
(≥69 vs.<69)	0.521(0.147-1.856)	0.315		
DHA (nmol/mL)				
(≥308 vs.<308)	0.109(0.014-0.862)	0.036	0.146(0.015-1.391)	0.094

Table 5. Abbreviations: HR, hazard ratio; 95% CI, 95% confidence interval; BMI, body mass index; HGB, hemoglobin; FIGO, International Federation of Gynecology and Obstetrics; SCC-Ag, squamous cell carcinoma antigen; EPA, Eicosapentaenoic acid; DHA, Docosahexaenoic acid; DPA, Docosapentaenoic acid.

Table 6. Relationships between clinicopathological data and the different levels of pretreatment C20:5 in CC.

	**C20:5<46**	**C20:5≥46**	
**Characteristics**	**N=27**	**N=62**	***P***
Age (years)			
<60	18(66.7)	25(40.3)	0.022
≥60	9(33.3)	37(59.7)	
BMI (kg/m^2)			
<25	22(81.5)	29(46.8)	0.002
≥25	5(18.5)	33(53.2)	
Hypertension history			
yes	6(22.2)	19(30.6)	0.416
no	21(77.8)	43(69.4)	
Diabetes history			
yes	1(3.7)	10(16.1)	0.198
no	26(96.3)	52(83.9)	
Lymph nodes metastasis			
yes	8(29.6)	20(32.3)	0.806
no	19(70.4)	42(67.7)	
FIGO stage			
IB3	1(3.7)	5(8.1)	0.483

II	10(37.0)	28(45.2)	
III	16(59.3)	29(46.8)	
Pre SCC-Ag (ng/mL)			
≤2.5	1(3.7)	8(12.9)	0.347
>2.5	26(96.3)	54(87.1)	
Post SCC-Ag (ng/mL)			
≤2.5	21(77.8)	53(85.5)	0.559
>2.5	6(22.2)	9(14.5)	
Size(cm)			
<4	7(25.9)	19(30.6)	0.653
≥4	20(74.1)	43(69.4)	
CR achieved			
yes	15(55.6)	48(77.4)	0.037
no	12(44.4)	14(22.6)	
HGB (g/L)			
<110	8(29.6)	12(19.4)	0.286
≥110	19(70.4)	50(80.6)	

Table 6. Abbreviations: BMI, body mass index; HGB, hemoglobin; FIGO, International Federation of Gynecology and Obstetrics; SCC-Ag, squamous cell carcinoma antigen**;** C20:5, eicosapentaenoic acid, EPA.

6.1 Supplementary Figures

Figure 1.

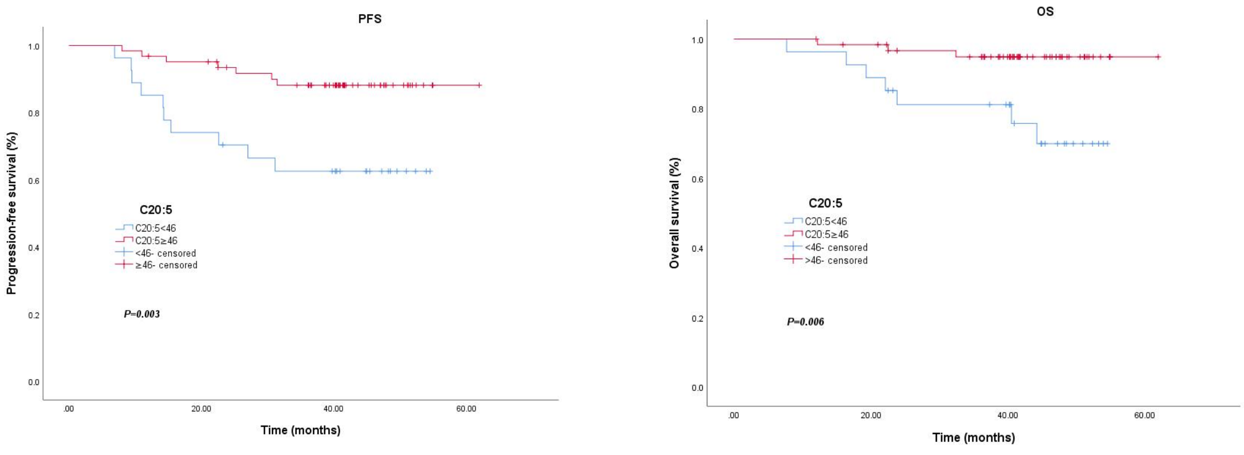

(1A) (1B)

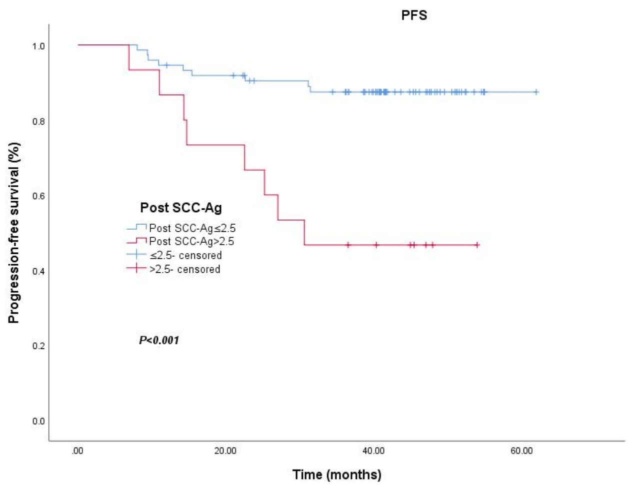

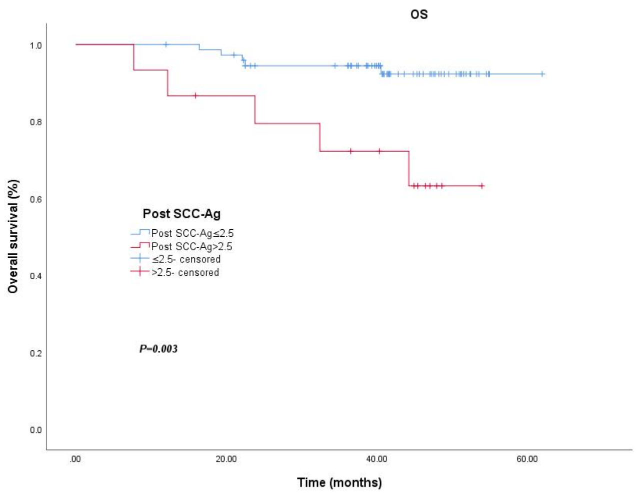

(1C)

(1D)

Figure 1. Kaplan–Meier curve for progression-free survival and overall survival for patients with low vs high C20:5 levels (1A) (*P*=0.003) and (1B) (*P*=0.006). Kaplan–Meier curve for progression-free survival and overall survival patients with low vs high post SCC-Ag levels (1C) (*P*<0.001) and (1D) (*P*=0.003). Abbreviations: Post SCC-Ag, posttreatment squamous cell carcinoma antigen; C20:5, eicosapentaenoic acid, EPA.

Figure 2.

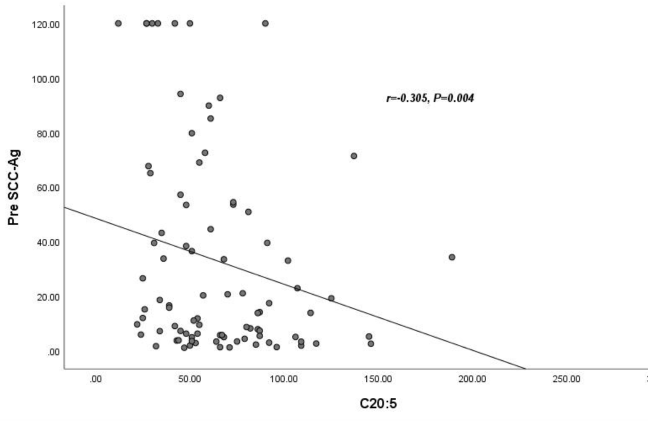

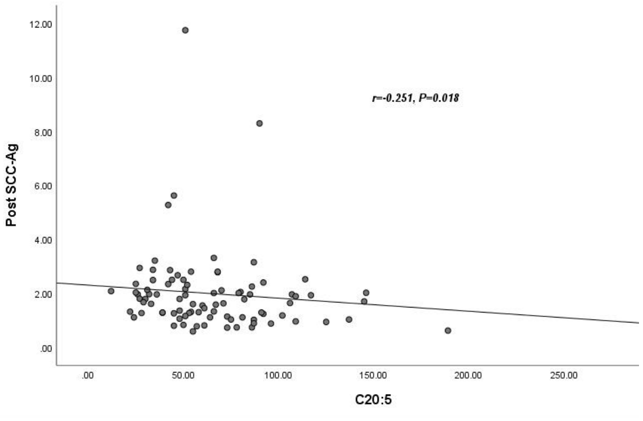

(2A)

(2B)

Figure 2. Correlation between plasma C20:5 and Post- SCC-Ag levels in 89 patients with CC. The plasma C20:5 levels were negatively correlated with pre SCC-Ag (r=−0.305, p=0.004) (2A). The plasma C20:5 levels were negatively correlated with post SCC-Ag levels (r=−0.251, p=0.018) (2B). Abbreviations: SCC-Ag, squamous cell carcinoma antigen; C20:5, eicosapentaenoic acid, EPA; Pre

SCC-Ag, pretreatment squamous cell carcinoma antigen; Post SCC-Ag, posttreatment squamous cell carcinoma antigen.

Figure 3.

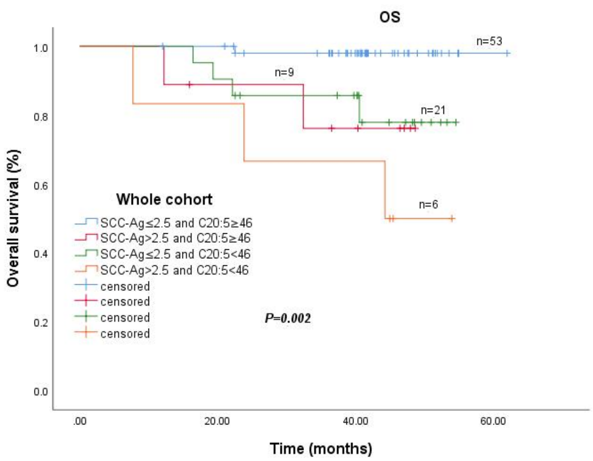

Figure 3. Abbreviations: SCC-Ag, squamous cell carcinoma antigen; C20:5, eicosapentaenoic acid, EPA.
